# Condition and size of the non‐native pikeperch *Sander lucioperca* (Linnaeus, 1758) in Portuguese river basins

**DOI:** 10.1002/ece3.7394

**Published:** 2021-04-06

**Authors:** João Gago, Ana Neves, Christos Gkenas, Diogo Ribeiro, Filipe Ribeiro

**Affiliations:** ^1^ Escola Superior Agrária – Instituto Politécnico de Santarém Quinta do Galinheiro – S. Pedro Santarém Portugal; ^2^ MARE Centro de Ciências do Mar e do Ambiente Faculdade de Ciências da Universidade de Lisboa, Campo Grande Lisboa Portugal

**Keywords:** condition factor, growth, non‐native fishes, phenotypic plasticity, pikeperch, *Sander lucioperca*

## Abstract

We studied life‐history traits focusing on the growth and condition of the pikeperch *Sander lucioperca* to evaluate its phenotypic plasticity when introduced to new environments. Pikeperch is a non‐native fish introduced to Iberian freshwater fauna in 1998 that quickly spread to other river basins through human‐mediated activities, occupying now a wide variety of habitats along mainland Portugal. Condition (*K* and SMI), fork length at age, and length–weight relationships were studied for Portuguese populations. Pikeperch fork length for ages 1, 2, 3, and 4 was different between several populations. We applied generalized linear models (GLM) to study the influence of habitat type, latitude, altitude, time after first detection, and fish prey richness on pikeperch populations size at age 4 and condition. We observed higher condition values on populations from lower altitudes at lentic systems more recently introduced. But higher fork length at age 4 was found in populations from higher altitudes, on older populations with higher prey richness. Habitat type, time since first detection, and fish fauna composition are discussed as the main environmental factors explaining the observed phenotypic plasticity with concerns on predatory impact on native fauna.

## Graphical abstract

*Sander lucioperca* is a non‐native fish which colonized a variety of Portuguese freshwaters and showed adaptive phenotypic plasticity on condition and growth to such new environments. Habitat type, time since first detection, and fish fauna composition are discussed as the main environmental factors explaining the observed phenotypic plasticity with concerns on predatory impact on native fauna.

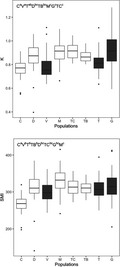

## 1 INTRODUCTION

The introduction of non‐native fishes has been shown to have significant deleterious effects on the freshwater ecosystems structure, functioning, and services (Reid et al., 2019), and for the biodiversity hotspot Mediterranean region, such impacts are already evident (Ribeiro & Leunda, 2012) and extensively described within the Iberian Peninsula (Leunda, 2010; Ribeiro et al., 2009). Despite being a recognized problem to freshwater conservation, non‐native fishes in Iberian Peninsula continue to increase, and consequently, this endemic rich area is considered a bioinvasion hotspot (Leprieur et al., 2008). Part of this non‐native fish richness is due to the broad environmental conditions that Iberian Peninsula exhibits, ranging from intermittent streams to high altitude mountainous areas, or karstic lakes (Sabater et al., 2009). This wide environmental range observed across Iberian Peninsula creates several opportunities to distinct non‐native fishes to establish wild populations but also constitutes an interesting challenge that may hamper their success (Amat‐Trigo et al., 2019; Ribeiro & Collares‐Pereira, 2010).

Biological responses to environmental variation are often measured by life‐history traits variation such as condition factor, body size, and growth patterns. Therefore, studying non‐native fish traits variation while invading new ecosystems and habitats might help to clarify how environment limits these invasive species success (Copp & Fox, 2007; Ribeiro & Collares‐Pereira, 2010). In fact, large‐scale intraspecific variation in growth and reproduction traits was previously described for several native and non‐native European freshwater fishes across latitudinal and environmental gradients (Blank & Lamouroux, 2007; Cucherousset et al., 2009; Lappalainen et al., 2008). Altitude related habitat characteristics seem to influence native fish body condition in Iberian rivers (Maceda‐Veiga et al., 2014), and for invasive species, age of the population (time after first detection), latitude, and temperature can also change considerably some life‐history traits throughout colonization, establishment, and dispersion (Bøhn et al., 2004; Copp & Fox, 2007; Gutowsky & Fox, 2012). Although several studies addressed this issue on non‐native fishes, mostly were done in small sized fish, generally invertivores with high life‐history plasticity (e.g., Gutowsky & Fox, 2012). More research is lacking on long‐lived fish and predators, which might have lesser capacity to adapt given their higher energetic demands, and the higher current rates of introduction of predatory fishes (Anastácio et al., 2019).

Pikeperch *Sander lucioperca* (Linnaeus, 1758) is a predatory fish native to central Europe and western Asia that has been introduced to European countries (Kottelat & Freyhof, 2007). This species was introduced to the Iberian Peninsula in the 1970s on Catalonian reservoirs (Miñano et al., 2002) and in 1998 was recorded to mainland Portugal (Barros et al., 2000). Nowadays, pikeperch has been established in most of the Iberian watersheds where it has important angling and commercial interest (Ribeiro, Gante, et al., 2009).

Size, somatic growth, and condition have been studied for several pikeperch populations both within their native (Kangur & Kangur, 1996; Keskinen & Majormäki, 2003; Ložys, 2004) and invaded ranges (Argillier et al., 2012; Nolan & Britton, 2018) and correlated with environmental gradients. For instance, pikeperch seems to grow faster, mature earlier, and present a shorter life span in lower‐latitude populations (Blank & Lamouroux, 2007) but Nolan and Britton (2018) did not find such linear relation. In fact, information about species trait variability in invaded areas is still limited, and studies performed in Iberian populations are scarce (Pérez‐Bote & Roso, 2012).

Therefore, given the recently established pikeperch populations across Iberian Peninsula and the wide extend of environmental gradients present in this region, assessing traits variability of this predatory fish along this environmental gradient can offer new insights into the biological mechanisms that lead to invasion success (Ribeiro, Gante, et al., 2009; Sabater et al., 2009).

Hence, the present study aims to evaluate the effects of latitudinal and altitudinal gradients, habitat type (lentic vs. lotic), time since first detection, and resource use (prey richness) on an array of non‐native predator biological traits (condition, length at age, and on length–weight relations) across Portuguese watersheds.

## 2 MATERIALS AND METHODS

### 2.1 Sampling and laboratory procedures

**FIGURE 1:**
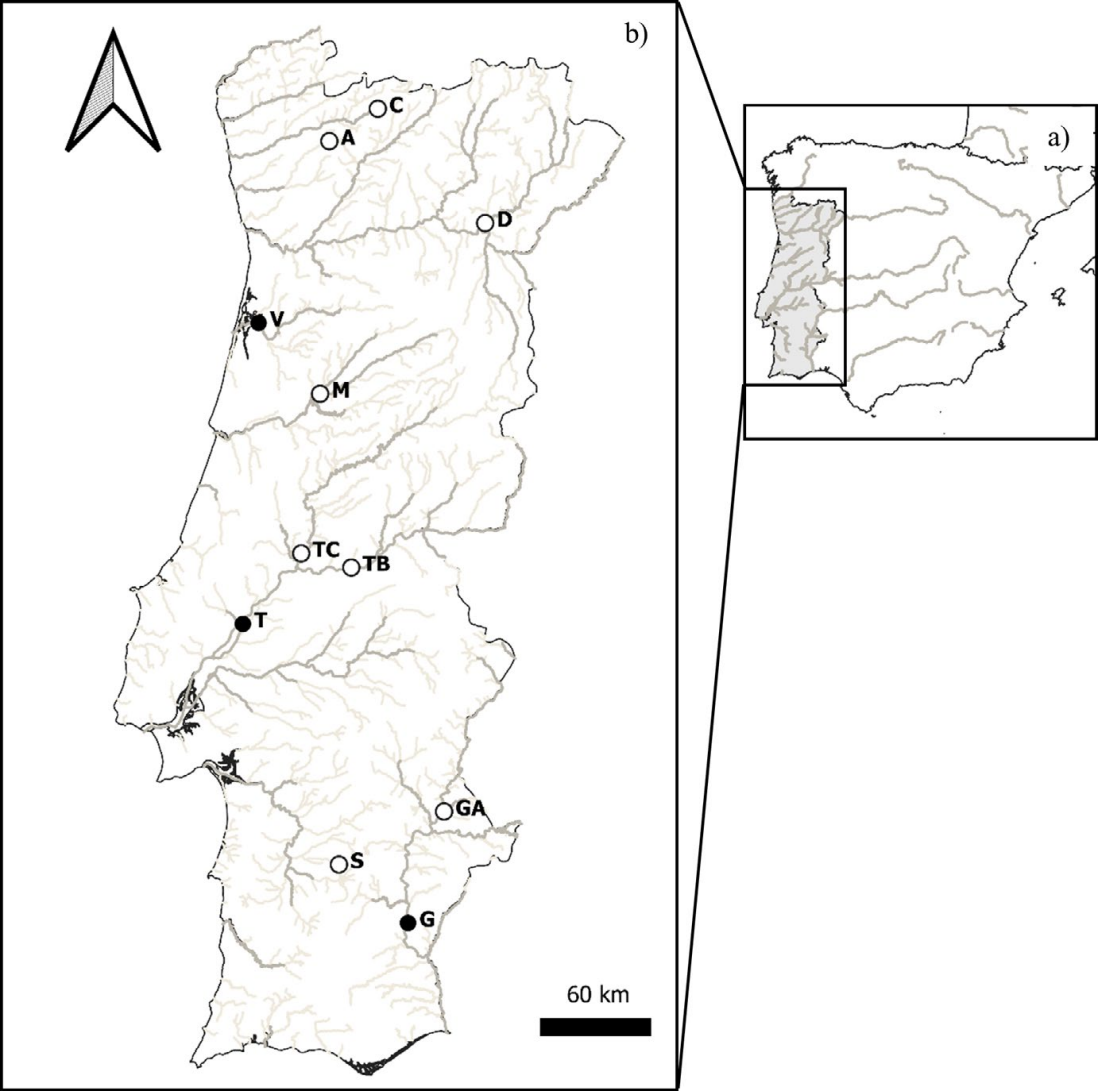
Map showing pikeperch (*Sander lucioperca*) sampled populations (a—Iberian Peninsula; b—mainland Portugal). Black circles correspond to lotic populations and white ones to lentic populations. From north to south (Drainage—Location/Habitat): C, Cávado River—“Alto do Rabagão” reservoir; A, Ave River—“Ermal”—reservoir; D, Douro River—“Foz do Sabor” reservoir; V, Vouga River—lotic section near “Angeja”; M, Mondego River—“Aguieira” reservoir; TC, Tagus River—“Castelo de Bode” reservoir; TB, Tagus River—“Belver” reservoir; T, Tagus River—lotic section near “Santarém”; S, Sado River—“Penedrão” reservoir; GA, Guadiana River—“Alqueva” reservoir; G, Guadiana River—lotic section near “Mértola”

Pikeperch were sampled during 2017 and 2018, from April to October in selected river basins either in lotic or lentic habitats (Figure 1) covering 11 populations across mainland Portugal. These populations were chosen because we wanted to cover, as broad as possible, the continental area of Portugal, with its environmental ranges, and are sites where there is considerable commercial fishing pressure to pikeperch which provided us easy access to fishes. In all sites, the main fishing technique consisted of overnight gillnets 80–150 mm mesh size. Some juveniles were also captured by standardized electrofishing (300–500 V, 1–5 A).

In the laboratory, specimens were measured (Fork Length – FL, to nearest 1 mm) and weighed (Eviscerated Weight – EW, to the nearest 0.01 g). Since not all populations were sampled at the same time, we used the eviscerate weight to avoid the influence of the gonad size and stomach fullness, giving more reliable values for the condition of the fish. When possible, sex was determined by gonad macroscopic examination.

As a simple, expedite and common methodology for pikeperch age estimation (e.g., Argillier et al., 2012; Nolan & Britton, 2018; Pérez‐Bote & Roso, 2012) we removed around 10 scales above the lateral line and below the anterior part of the dorsal fin that were posteriorly cleaned and mounted on microscopic slides. Selected scales were photographed under a binocular lens and examined using freeware Fiji image analysis program. Three experienced independent readers determined the individual age on the same scale. Scales with age readings different among the readers were discarded and only those that had the same age reading from at least two readers were considered valid (91.4% of the cases). The possible age underestimation, using scales, for the low number of larger and older pikeperch was minimized by the relatively low maximum age found (9 years) when compared to maximum longevity of 17 years (Kottelat & Freyhof, 2007) and maximum ages found in other studies (14 years old for Argillier et al. (2012), and 11 years for Nolan and Britton (2018)).

### 2.2 Population characterization

For each pikeperch population, data were extracted from a set of environmental features to evaluate their effect on trait variability (Table 1). Each population was classified based on its main habitat type (lotic/lentic). As a surrogate of water temperature, mean annual air temperature (ºC) data were selected from Instituto Português do Mar e da Atmosfera (IPMA) site (http://portaldoclima.pt/) considering the historical 1971–2000 period for the nearest meteorological station. Latitude, longitude, and altitude were obtained on Google Earth^®^ (Google Inc.). The first year that pikeperch were detected in each basin was determined from data obtained in literature (see Ribeiro, Gante, et al., 2009), fishing blogs and fora as well as from anglers' and professional fishermen information given in interviews. With precautionary methodologies, these resources (interviews, online blogs, and forums) have already proved to be accurate to estimate introduction and spread of non‐native freshwater fish fauna in Portugal (Banha et al., 2015; Gago et al., 2016; Martelo et al., 2021). As a surrogate of food resources, the fish prey richness (FPR) was determined as the maximum number of fish prey species found in stomach content analysis performed on the same fish populations (Ribeiro, 2017).

**TABLE 1:** Descriptive values for each pikeperch population (*Sander lucioperca*) considering habitat type (lentic or lotic), latitude (ºN), longitude (ºW), altitude (m), temperature (ºC ‐ mean air temperature from the 1971–2000 period), invasion year (year of first record), and fish prey richness (FPR) found by Ribeiro (2017)

Population acronyms	Habitat	Lat.	Long.	Alt.	Temp (°C)	Year	FPR
C	Lentic	41.75	−7.81	862	12.8	2006	3
A	Lentic	41.59	−8.13	330	12.2	1998	1
D	Lentic	41.18	−7.11	105	11.7	1999	5
V	Lotic	40.69	−8.59	4	14.1	2011	6
M	Lentic	40.34	−8.19	124	13.6	2008	2
TC	Lentic	39.55	−8.31	119	14.4	2012	6
TB	Lentic	39.48	−7.99	45	14.4	2004	3
T	Lotic	39.2	−8.68	4	15.8	2008	6
GA	Lentic	38.27	−7.41	90	15.6	2005	3
S	Lentic	38.01	−8.07	201	16.3	2012	3
G	Lotic	37.72	−7.64	7	16.3	2009	5

Note

Latitude and Longitude coordinates are in WGS84 in decimal fraction. Population acronyms as the ones in Figure 1. C, Cávado River—“Alto do Rabagão” reservoir; A, Ave River—“Ermal”—reservoir; D, Douro River—“Foz do Sabor” reservoir; V, Vouga River—lotic section near “Angeja”; M, Mondego River—“Aguieira” reservoir; TC, Tagus River—“Castelo de Bode” reservoir; TB, Tagus River—“Belver” reservoir; T, Tagus River—lotic section near “Santarém”; S, Sado River—“Penedrão” reservoir; GA, Guadiana River—“Alqueva” reservoir; G, Guadiana River—lotic section near “Mértola.”

As life‐history traits of each population we estimated the Fork Length at each age, the fish condition characterized by condition factor (*K*) calculated according with Anderson and Gutreuter (1983): *K* = 10^5^ Weight × Length^−3^, and Scaled Body Mass Index (SMI) according to Maceda‐Veiga et al. (2014): $\text{SMI}=W_{i}{\left[L_{0}/L_{i}\right]}^{b_{\text{SMA}}}$, where *W_i_* and *L_i_* are the weight and length of each specimen, respectively, *L*_0_ is a suitable length to which the condition values are standardized, and *b*_SMA_ is the scaling exponent of the mass–length relationship. The Fork Length arithmetic mean for the entire pikeperch populations (32.8 cm), as the suitable length to which the condition is standardized (*L*_0_), was used for SMI calculations.

The length–weight relationships, EW = *a* FL*^b^*, were also estimated for each population.

### 2.3 Data and statistical analysis

Differences on Fork Length at age, among pikeperch populations, were analyzed using Kruskal–Wallis test for each age separately. Only populations with more than five observations were included which excluded plus 5 years age classes. Kruskal–Wallis tests were also performed on *K* and SMI. Posterior multiple comparisons were evaluated with Conover post hoc tests, with Bonferroni correction for multiple comparisons.

Differences on length‐weight slopes (*b*) between each pair of populations were investigated by Student's *t* test. Deviations of sex ratio (females:males) from 1:1 were assessed with chi‐square tests.

Generalized linear models (GLM) were used to test the effect of habitat, latitude, altitude, temperature, time of first detection (redefined as the number of years that the population is known to exist in each locality) and fish prey richness on pikeperch biological traits (FL at age 4 and the two condition indices). Supported by the proportions of mature pikeperch among ages (see results), length at age 4 was considered a proxy of pikeperch juvenile growth. Age 4 as the onset of maturity seems also concordant with Kottelat and Freyhof (2007) and reliable with the variation found in European populations (Lappalainen et al., 2003). Data were standardized to assure comparable scales, and predictive variables were tested for multicollinearity using the variance inflation factor (VIF) estimated with mctest R package (Imdadullah et al., 2016; Ullah & Aslam, 2018; Ullah et al., 2019). The two correlated variables, latitude, and temperature, with VIF values above 5 were excluded from the generalized linear models (see Appendix S1). All statistical analyses were implemented in RStudio (R Core Team, 2020). For the above referred Data and Statistical analysis, we excluded three populations with less than 30 individuals (A—Ave; GA—Guadiana Alqueva; S—Sado).

No sex differentiation was made in this study for fork length and length–weight relations due to limitations on sampling size and because several pikeperch were still immature or in resting reproduction phase when caught. Yet, Kangur and Kangur (1996) and Pérez‐Bote and Roso (2012) found no differences between the growth rates of males and females pikeperches and in length–weight relations between sexes (Pérez‐Bote & Roso, 2012). Equally, sexes were combined in condition factor calculations as performed by Kangur and Kangur (1996), Ložys (2004) and Argillier et al. (2012).

## 3 RESULTS

Overall 11 populations were analyzed encompassing a total of 383 individuals (Table 2). In the Douro, Vouga, Mondego, and lotic Tagus populations, most of the individuals were still immature due to younger modal age class found at those locations. In fact, when considering all the pikeperch populations, nearly 55% of the analyzed specimens were younger than 4 years and about 95% of these were non‐reproductive fish. Conversely, around 60% of the fish with 4 years of age were mature when we collected samples during the spawning season (April to July) and beyond age 4 all pikeperch were mature. Sex ratio did not present any bias toward females or males (Kruskal–Wallis, *p* > 0.05).

**TABLE 2:** Sample characterization of pikeperch (*Sander lucioperca*) populations considering number of individuals sampled (*N*), Mean fork length in cm (FL), Sex ratio as proportion of Females:Males (F:M) and number of immature fishes in brackets, mean condition factor (*K*), mean scaled body mass index (SMI), and modal age class (Age)

Population acronyms	*N*	FL (min‐max)	Sex ratio (F:M) (Immature)	*K* (min‐max)	SMI (min‐max)	Age (min‐max)
C	30	35.3	0.5:1	0.761	267	3
		23.7–58.0	(10)	0.550–0.913	198–319	2–6
A	12	44.2	1.4:1	0.761	260	4
		37.7–55.4	(0)	0.678–0.856	231–294	4–6
D	40	30.3	2.67:1	0.871	311	3
		13.8–43.4	(29)	0.396–1.06	141–385	0–7
V^a^	36	20.4	1.7:1	0.788	299	1
		9.6–65.8	(28)	0.635–1.11	243–362	1–7
M	70	25.6	1.8:1	0.902	330	1
		16.3–53.5	(45)	0.663–1.10	241–415	1–7
TC	30	44.2	1.1:1	0.917	313	4
		29.3–53.2	(0)	0.809–1.05	272–357	2–6
TB	34	32.7	1.1:1	0.873	309	3
		18.4–42.9	(2)	0.802–0.979	278–347	1–4
T^a^	33	23.4	1.5:1	0.822	307	1
		8.6–62.3	(23)	0.634–1.11	250–394	0–7
GA	29	44.4	2.1:1	0.844	288	4
		34–54.8	(1)	0.715–0.946	240–325	3–7
S	29	36.5	2:1	0.934	328	3
		22.1–62.1	(8)	0.797–1.20	278–439	2–6
G^a^	40	42.2	1.2:1	0.917	315	4
		24.3–72.4	(3)	0.592–1.28	205–413	2–9

Note

Minimum (min) and maximum (max) values for FL, *K*, SMI, and age are also given. Population acronyms as the ones in Figure 1.

^a^ Represents populations from lotic habitats, and all the others are from lentic systems.

**FIGURE 2:**
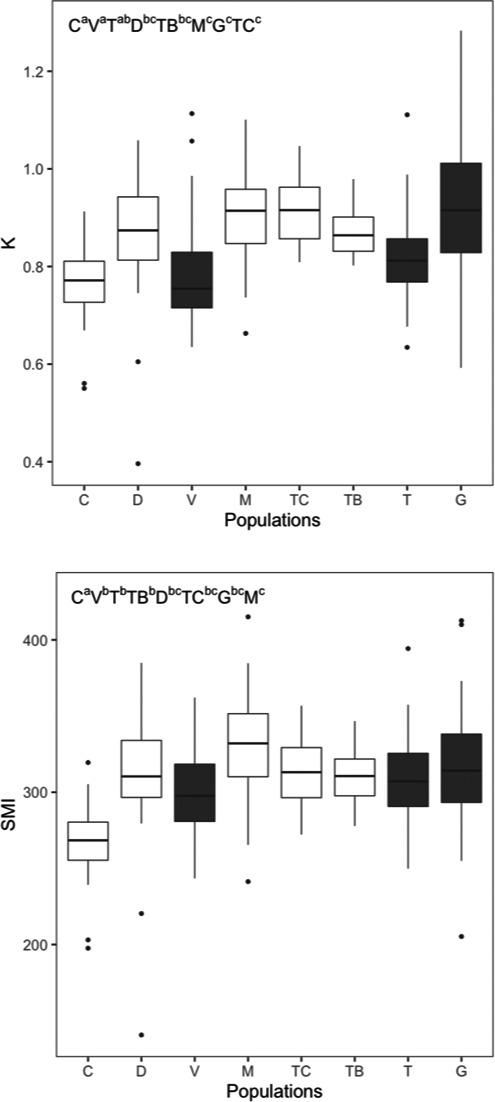
Boxplots for *K* condition factor (above) and Scaled Mass Index (SMI) (below) of the eight studied populations (populations acronyms as the ones in Figure 1) displayed from North (left) to South (right). Boxplots in white represent lentic populations and in black riverine populations. The box represents the interquartile range (IQR; 25th and 75th percentiles), and the line within the box is the median. Whiskers represent the 75th percentile � 1.5 × IQR and the 25th percentile � 1.5 × IQR. Data beyond the end of the whiskers are outliers and plotted as points. On the upper left corners, different letters above populations acronyms show the significant differences on condition factors among populations (Conover test, *p* > 0.05)

Fish condition varied significantly among populations (Kruskal–Wallis, *p* < 0.0001 for both *K* and SMI) and was lower in the northern basins (Figure 2). For instance, pikeperch mean *K* condition for the northern lentic populations (Cávado) was 0.761, while in the two southern populations (Guadiana lotic and Tagus TC reservoir) was 0.917. Similar trend was observed for SMI with the lowest value observed in Cávado (267), while the highest value was found in Mondego lentic populations (330). Parameters from the length‐weight relations are shown in Table 3 (see also Appendix S2). The lotic populations from the Guadiana and Vouga rivers had considerable higher positive allometric growth (*t* test, *p* < 0.05) than all the other populations but some more pairwise differences were found (Appendix S3).

**TABLE 3:** Regression parameters *a* and *b* (curve slope ± standard error [*SE*]) from the length‐weight equation EW = *a* FL*^b^*

Population acronyms	*a* (10^–3^)	*b* ± *SE*
C	4.05	3.18 ± 0.07
D	7.47	3.05 ± 0.13
V^†^	1.98	3.41 ± 0.03
M	5.68	3.14 ± 0.04
TC	5.49	3.13 ± 0.15
TB	10.99	2.94 ± 0.09
T^†^	4.03	3.21 ± 0.05
G^†^	1.463	3.48 ± 0.09

Note

Population acronyms as the ones in Figure 1.

^†^ Represents populations from lotic habitats, and all the others are from lentic systems.

**FIGURE 3:**
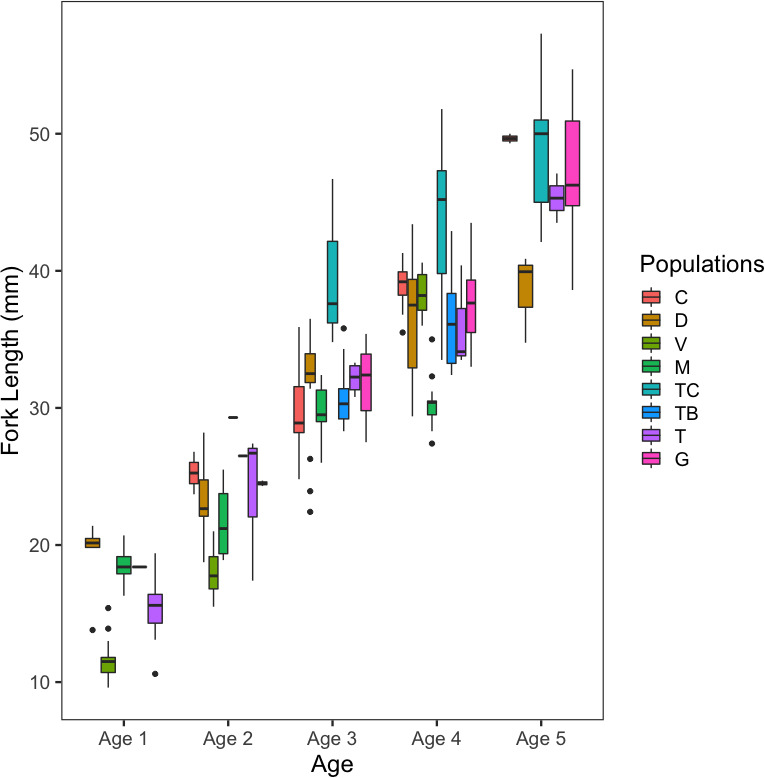
Boxplots for Fork Length (mm) at age (1–5 years old) of the eight studied populations (populations acronyms as the ones in Figure 1) displayed from North (left) to South (right). The box represents the interquartile range (IQR; 25th and 75th percentiles), and the line within the box is the median. Whiskers represent the 75th percentile � 1.5 × IQR and the 25th percentile � 1.5 × IQR. Data beyond the end of the whiskers are outliers and plotted as points

Age composition was similar between most of the populations (Figure 3 and Appendix S4), being mostly composed by ages 3, 4, and 5, but not all age classes were represented (with *n* ≥ 5) at all sites (Table 4). Length at ages 1 (Kruskal–Wallis, *p* < 0.0001) and 2 (Kruskal–Wallis, *p* < 0.001) showed significant differences among most of the compared populations, while no differences were found at age 5 (Kruskal–Wallis, *p* = 0.08671). For age 3 (Kruskal–Wallis, *p* < 0.05), only the Douro population showed to have longer pikeperch than Cávado (Conover, *p* < 0.05), Mondego (Conover, *p* < 0.005) and Tejo‐Belver (Conover, *p* < 0.05) populations. For age 4 (Kruskal–Wallis, *p* < 0.0001), Mondego contained significantly shorter fish than all the other populations (Conover, *p* < 0.005) but pairwise differences were also found between several other basins.

**TABLE 4:** *Sander lucioperca* mean fork length (mm) ± standard deviation for ages 1–5

Age	1	2	3	4	5
Cávado (C)	\	\	29.8 ± 3.44	38.9 ± 1.99	\
Douro (D)	19.3 ± 2.75	23.1 ± 2.38	32.0 ± 2.77	36.6 ± 4.23	\
Vouga (V)^†^	11.6 ± 1.33	18.0 ± 1.87	\	38.3 ± 1.80	\
Mondego (M)	18.5 ± 1.00	21.7 ± 2.66	29.9 ± 1.55	30.4 ± 1.62	\
Tejo ‐ C. Bode (TC)	\	\	\	43.5 ± 5.45	48.6 ± 4.45
Tejo ‐ Belver (TB)	\	\	30.7 ± 2.09	36.3 ± 3.51	\
Tejo (T)^†^	15.4 ± 2.02	\	\	\	\
Guadiana (G)^†^	\	\	31.9 ± 2.94	37.9 ± 3.24	47.0 ± 5.34
Kruskal–Wallis test	V^a^T^b^M^c^D^c^	V^a^M^b^D^b^	M^a^C^ab^TB^ab^G^ab^D^b^	M^a^TB^b^D^b^G^bc^V^bcd^C^bcd^TC^d^	TC^a^G^a^

Note

Populations with significant differences (Kruskal–Wallis test, *p* < .05) are signaled with different letters at the top of the acronyms. Population acronyms as the ones in Figure 1.

^†^ Represents populations from lotic habitats, and all the others are from lentic systems.

The GLM results (Table 5) displayed that altitude and year of introduction were significant predictors in all the three models. Habitat type was significant for both fish condition indices but not for Fork Length at age 4, while fish prey richness (FPR) was a significant predictor only for length at age 4. Condition (either *K* and SMI) increased with decreasing altitude and showed to be higher at lentic systems and in recently invaded areas. Pikeperch length at age 4 increased with increasing altitude and also showed to have positive relations with the time since detection and prey richness.

**TABLE 5:** Summary table with the estimated regression parameters, standard errors, *t*‐values, and *p*‐values for the generalized linear model applied to fork length at age 4 (FL at age 4), condition factor (*K*), and Scaled Body Mass Index (SMI)

Variable	FL at age 4				*K*				SMI			
	Estimate	*SE*	*t*	*p*	Estimate	*SE*	*t*	*p*	Estimate	*SE*	*t*	*p*
Intercept	37.94	0.51	74.7	***	0.89	0.01	104.2	***	314.2	2.64	119.1	***
Altitude	1.25	0.44	2.89	**	−0.04	0.01	−6.52	***	−16.54	1.98	−8.37	***
Year	−0.14	0.46	−0.29		−0.02	0.01	−2.94	**	−3.67	2.05	−1.79	
Habitat	−2.36	1.14	−2.08	*	−0.07	0.02	−4.02	***	−12.48	5.64	−2.21	*
FPR	3.80	0.47	8.07	***	0.01	0.01	0.87		−6.69	2.35	−2.85	**

Note

Significance codes: *p* < 0.05 (*); *p* < 0.01 (**); *p* < 0.001 (***).

## 4 DISCUSSION

In this study, pikeperch exhibited a wide variability on growth and condition parameters which seems to be influenced by environmental variables and population age. Considerable intraspecific variability of non‐native fishes' traits was previously observed in Iberian freshwater environments but were focused on short‐lived invertivores fish (Amat‐Trigo et al., 2019; Ribeiro & Collares‐Pereira, 2010), and there was scarce information about non‐native predator biological trait variation as response to local invasion. In fact, the high trait variability of the predator pikeperch suggests a high adaptive capacity to local environmental conditions and, consequently, broad invasion potential across Iberian watersheds (Ribeiro, Collares‐Pereira, et al., 2009).

Freshwater fish body size is associated with various individual characteristics since many physiological rates such as respiration, reproduction, or growth are size‐dependent (Benejam et al., 2018). Pikeperch in good condition may be assumed to have higher growth rate, thus body size and condition are good growth indicators of local adaptation (Ložys, 2004). The present study showed that each population seems to present a balance between fish condition and juvenile length, suggesting different strategies in order to cope with local environmental conditions and available resources.

Overall, the two condition factors produced similar results, and *K* values fall within the range presented in the literature (Argillier et al., 2012; Kangur & Kangur, 1996; Ložys, 2004). Similarly, growth variations reflected on length at specific ages have already been noticed among pikeperch populations from the same country (Argillier et al., 2012 and the references therein).

Higher condition was found among pikeperch populations from lower altitudes. Maceda‐Veiga et al. (2014) found higher SMI in higher altitudes but this study focused on fish inhabiting mountainous areas, belonging to the minnows family (Cyprinidae) or the trout family (Salmonidae). Yet, pikeperch preferentially inhabits large rivers and eutrophic lakes generally found at lower altitudes (Keskinen & Majormäki, 2003), which is consistent with our observed condition patterns. The results obtained here showed that better pikeperch condition is found in more recently invaded lentic habitats, supporting that more stable local conditions of such artificial water bodies enable a better population establishment. Conversely, longer pikeperch length at age 4 was found in older populations from higher altitude sites that were generally thinner (with lower *K* and SMI values). These are lentic populations which present lower fish diversity but are mostly composed by pikeperch fish preys (Ribeiro, 2017), enabling a faster growth. Clavero et al. (2013) previously showed that high altitude reservoirs in Iberian Peninsula present lower non‐native fish richness, while lowland lotic environments generally present high fish diversity (Filipe et al., 2010). This is consistent with the higher FPR in lotic populations (Ribeiro, 2017) leading to more bulky fish in lotic systems, as observed by the significant higher value for b slope of the length‐weight equations, suggesting high biomass input relative to fish length. Besides higher prey richness, such lotic systems may have less intraspecific competition related to the younger age of the population and the more intense fishing pressure from professional fishermen.

Previous work described a temperature effect on pikeperch growth (Keskinen & Majormäki, 2003; Lehtonen et al., 1996; Ložys, 2004) which is negatively related to latitude in the northern hemisphere. Pikeperch seems to grow faster, mature earlier and display a shorter life span in lower‐latitude populations (Blank & Lamouroux, 2007). In this study, we also found the negative correlation between latitude and temperature, but due to GLM assumptions, we could not detect the effect of such variables on both condition and juvenile growth. Nevertheless, both condition indices proved to be significant higher on southern populations, but such trend was indistinguishable for pikeperch length attained at age 1–5. Copp and Fox (2007), for *Lepomis gibbosus*, found that juvenile growth rate appears to decrease significantly with increasing latitude and this tendency seems to extend into adult stage (Cucherousset et al., 2009). However, Lappalainen et al. (2008) for *Rutilus rutilus* did not find a linear relation of the von Bertallanfy growth parameters with latitude as well as Nolan and Britton (2018) for pikeperch. Yet, Nolan and Britton (2018) review encompass a larger latitude range across native and invasive regions and uses different growth parameters based on von Bertallanfy growth curves derived from both in situ determination and literature research, while current study followed the same approach for all the pikeperch populations. Introduction year influenced condition and length at age 4 with shorter fish but heavier on more recent invaded habitats. It is expected that freshwater fishes during the process of invasion to experience alterations in life‐history traits. For instance, Bøhn et al. (2004) documented lesser growth on the later stages of *Coregonus albula* invasion due to higher density and resource competition and Gutowsky and Fox (2012) also found significant differences in somatic growth for the round goby (*Neogobius melanostomus*) between the area it was first introduced and the edges of its expanding range. Pikeperch populations in Portugal not seemed to support the rapid growth in length pattern in younger populations but instead it was observed an increase in fish body condition. Such pattern could reflect the pioneer strategy to favor reproduction instead of growth in still low population density, provided by high availability in prey resources at the beginning of the invasion of this predatory fish. In fact, the downstream reaches of main studied rivers were only recently invaded in comparison with several lentic populations found inland, and the higher prey availability found in these areas might strengthen this effect (Ribeiro, Gante, et al., 2009).

The GLM analyses detected the effect of habitat type (lentic vs. lotic) on condition factors with better condition found in lentic populations, but no effect of this variable was detected for length at age 4. The effect of habitat type in life‐history traits within European freshwater species due to contrasting environmental stability was already detected by Blank and Lamouroux (2007).

Food availability is also considered to be one of the most important factors influencing growth rate in freshwater fish and Lehtonen et al. (1996) already proved it for pikeperch. Ribeiro (2017) pointed out the opportunistic feeding behavior of pikeperch according to prey availability, so potential variations in prey use might also explain the effect of habitat type, because higher number of fish prey were found on lotic sections.

The enlarged sampling period and dependence on commercial fishing and angling are also common on other pikeperch growth studies (Nolan & Britton, 2018; Pérez‐Bote & Roso, 2012). These methodology constraints, as for example number of sampling years, sampling season, and parameter estimation, have already been referred as influencing the estimation of many European freshwater fish life‐history traits (Blank & Lamouroux, 2007). Furthermore, the intense fishing pressure applied to pikeperch populations, mainly targeted to larger individuals, may also influenced our results as for instance not much older pikeperch were sampled.

Taken together, the relative phenotypic variability presented by this predatory fish, despite observed in other invasive fish species introduced to Iberian, freshwater systems, mostly small sized and omnivorous (e.g., Almeida et al., 2009; Amat‐Trigo et al., 2019), are relatively new since it was not expected such plasticity on a top predator. Other invasive predators like the largemouth bass *Micropterus salmoides* showed limited plasticity when invading Mediterranean systems with invasion difficulties for lotic habitats (Ribeiro & Collares‐Pereira, 2010). In fact, pikeperch is a highly successful invader occupying a wide variety of habitats and being the most widespread predatory non‐native fish that in about 20 years invaded most of the Portuguese drainages (Martelo et al., 2021; Ribeiro, Gante, et al., 2009). Variations found in pikeperch biological traits suggest adaptation to changes in environmental factors but may show some capacity to respond to management actions such as unrestricted fisheries. This will represent a management challenge in a region with high number of endemic fish, many threatened and highly susceptible to these invasive top predators, once its original fish communities in Iberian Peninsula are devoid of any native predator.

## CONFLICT OF INTEREST

The authors declare no conflict of interest.

## AUTHOR CONTRIBUTION

**João Gago:** Conceptualization (equal); Data curation (equal); Formal analysis (equal); Investigation (equal); Methodology (equal); Supervision (equal); Validation (equal); Visualization (equal); Writing‐original draft (equal); Writing‐review & editing (equal). **Ana Neves:** Data curation (equal); Methodology (equal); Software (equal); Writing‐original draft (equal); Writing‐review & editing (equal). **Christos Gkenas:** Conceptualization (equal); Data curation (equal); Methodology (equal); Writing‐original draft (equal); Writing‐review & editing (equal). **Diogo Ribeiro:** Investigation (equal); Methodology (equal); Writing‐review & editing (equal). **Filipe Ribeiro:** Conceptualization (equal); Formal analysis (equal); Funding acquisition (equal); Investigation (equal); Project administration (equal); Resources (equal); Validation (equal); Writing‐original draft (equal); Writing‐review & editing (equal).

## Supporting information

Appendix S1‐S4

## Data Availability

The data that support the findings of this study are available at Dryad digital repository (https://doi.org/10.5061/dryad.bnzs7h49m).
