# Supplementary material for: Condition and size of the non‐native pikeperch Sander lucioperca (Linnaeus, 1758) in Portuguese river basins
Source: Ecol Evol. 2021 Apr 6;11(10):5065–74. doi: 10.1002/ece3.7394 (PMC8131815; doi:10.1002/ece3.7394)

**Appendices**

**Appendix 1.**

1. Correlation between GLM variables (Lat – Latitude; Alt – Altitude; Temp – Temperature; Year - Number of years that the population is known to exist in each locality; FPR – Fish Prey Richness.


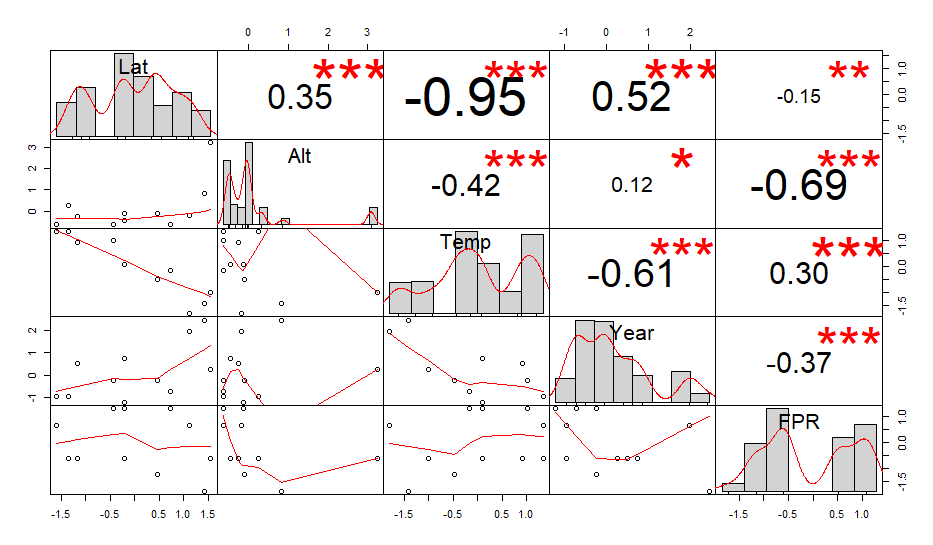


1. Variance Inflation Factor (VIF) for Latitude (Lat); Altitude (Alt); Temperature (Temp); Number of years that the population is known to exist in each locality (Year); Fish Prey Richness (FPR); and Habitat

| **Variable** | **VIF** |
| --- | --- |
| Lat | 10.72 |
| Alt | 1.95 |
| Temp | 17.40 |
| Year | 2.55 |
| FPR | 1.92 |
| Habitat | 4.26 |

**Appendix 2.** Fork Length – Eviscerated Weight relations for the 11 studied *Sander lucioperca* populations.


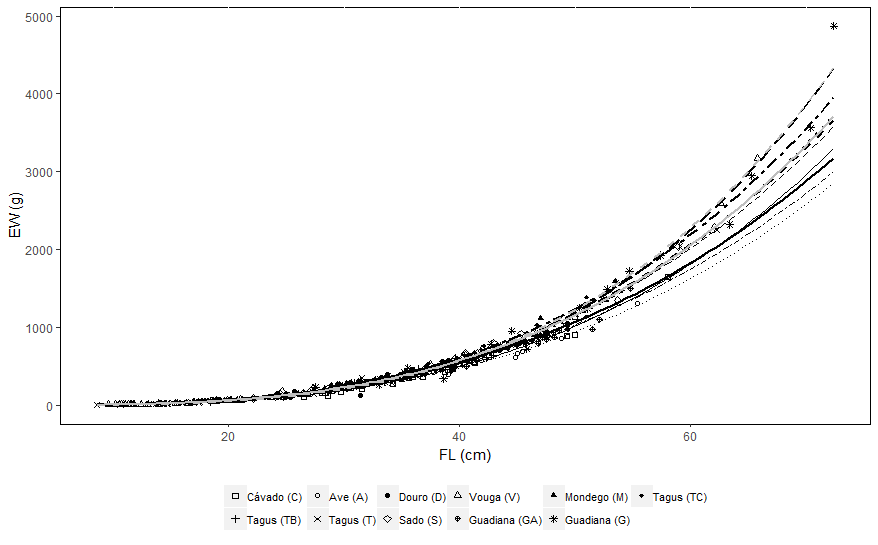


Light black solid line: **Cávado (C)** (EW = 0.004052 × FL^3.178^); light black dotted line: **Ave (A)** (EW = 0.007837 × FL^2.991^); light black long dash line: **Douro (D)** (EW = 0.007473 × FL^3.054^); grey dashed line: **Vouga (V)** (EW = 0.0019772 × FL^3.410^); black two dash line: **Mondego (M)** (EW = 0.0056835 FL^3.141^); grey solid line: **Tejo – Castelo do Bode** **(TC)** (EW = 0.005486 × FL^3.135^); black solid line: **Tejo – Belver (TB)** (EW = 0.010989 × FL^2.936^); black dotted line: **Tejo (T)** (EW = 0.0040256 × FL^3.210^); light black two dash line: **Guadiana – Alqueva (GA)** (EW = 0.01379 × FL^2.870^); black dashed line: **Sado (S)** (EW = 0.007596 × FL^3.056^); black long dash line: **Guadiana (G)** (EW = 0.001463 × FL^3.480^).

**Appendix 3**. Comparison results for median FL at age (bellow diagonal) and FL vs EW relationship slope – b (above diagonal). Only the ages at which occurred significant differences in FL (Conover test, p < 0.05) are signaled. For slope results only significant differences are signaled (t-test, p < 0.05). **^†^** represents populations from lotic habitats and all the others are from lentic systems.

|  | **C** | **D** | **V ^†^** | **M** | **TC** | **TB** | **T ^†^** | **G ^†^** |
| --- | --- | --- | --- | --- | --- | --- | --- | --- |
| **Cávado (C)** |  |  | b |  |  | b |  | b |
| **Douro (D)** | 3 |  | b |  |  |  |  | b |
| **Vouga (V) ^†^** |  | 1,2 |  | b | b | b | b |  |
| **Mondego (M)** | 4 | 3,4 | 1,2,4 |  |  | b |  | b |
| **Tagus - C. Bode (TC)** |  | 4 |  | 4 |  |  |  | b |
| **Tagus - Belver) (TB)** |  | 3 |  | 4 | 4 |  | b | b |
| **Tagus (T) ^†^** |  | 1 | 1 | 1,4 |  |  |  | b |
| **Guadiana (G) ^†^** |  |  |  | 4 | 4 |  |  |  |

**Appendix 4.** Age-length relationships for the 11 studied pikeperch (*Sander lucioperca*) populations.


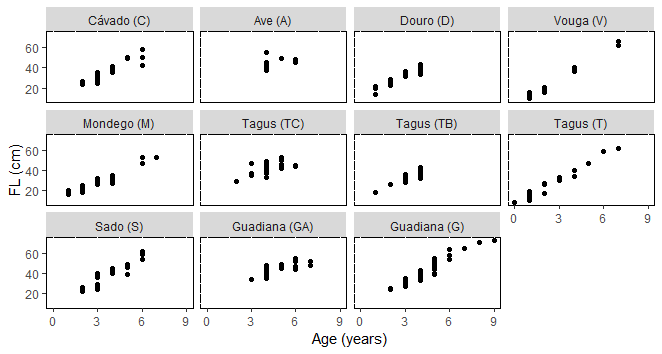

Supplement: Supplementary file 1 — Appendix S1‐S4 [file ECE3-11-5065-s001.docx]
